# Injectable gellan gum-based nanoparticles-loaded system for the local delivery of vancomycin in osteomyelitis treatment

**DOI:** 10.1007/s10856-015-5604-2

**Published:** 2015-11-30

**Authors:** Urszula Posadowska, Monika Brzychczy-Wloch, Elzbieta Pamula

**Affiliations:** Department of Biomaterials, Faculty of Materials Science and Ceramics, AGH University of Science and Technology, al. A. Mickiewicza 30, 30-059 Krakow, Poland; Department of Microbiology, Medical College, Jagiellonian University, ul. Czysta 18, 31-121 Krakow, Poland

## Abstract

Infection spreading in the skeletal system leading to osteomyelitis can be prevented by the prolonged administration of antibiotics in high doses. However systemic antibiotherapy, besides its inconvenience and often low efficacy, provokes numerous side effects. Thus, we formulated a new injectable nanoparticle-loaded system for the local delivery of vancomycin (Vanc) applied in a minimally-invasive way. Vanc was encapsulated in poly(l-lactide-*co*-glycolide) nanoparticles (NPs) by double-emulsification. The size (258 ± 11 nm), polydispersity index (0.240 ± 0.003) and surface potential (−25.9 ± 0.2 mV) of NPs were determined by dynamic light scattering and capillary electrophoresis measurements. They have a spherical morphology and a smooth topography as observed using atomic force microscopy. Vanc loading and encapsulation efficiencies were 8.8 ± 0.1 and 55.2 ± 0.5 %, respectively, based on fluorescence spectroscopy assays. In order to ensure injectability, NPs were suspended in gellan gum and cross-linked with Ca^2+^; also a portion of dissolved antibiotic was added to the system. The resulting system was found to be injectable (extrusion force 11.3 ± 1.1 N), reassembled its structure after breaking as shown by rheology tests and ensured required burst release followed by sustained Vanc delivery. The system was cytocompatible with osteoblast-like MG-63 cells (no significant impact on cells’ viability was detected). Growth of *Staphylococcus* spp. reference strains and also those isolated from osteomyelitic joints was inhibited in contact with the injectable system. As a result we obtained a biocompatible system displaying ease of application (low extrusion force), self-healing ability after disruption, adjustable drug release and antimicrobial properties.

## Introduction

The traditional way of pathogenic bacteria elimination from the musculoskeletal environment involves surgical removal of necrotic tissue, osseous repair and antibiotherapy applied for 3–6 weeks [1, 2]. If such treatment is not effective, there is a risk that chronic disease called osteomyelitis develops [1, 2]. Application of a glycopeptide antibiotic—vancomycin (Vanc) is advised for life-threatening staphylococci infections that are a dominating cause of osteomyelitis [3] and display resistance to typical first-line antibiotics [4, 5]. However, the very poor bioavailability of Vanc is an obstacle in the therapy, because it is a large hydrophilic molecule that is moved ineffectively across the lipophilic gastrointestinal mucosa [6]. As a result, high doses of Vanc are used that in general cause nephro- or ototoxicity, allergies and problems with the gastrointestinal tract [6].

To deal with such problems local, site-specific routes of Vanc delivery are being explored, e.g. based on calcium phosphate cements or pastes [7], coatings on titanium alloys [8], poly(methyl methacrylate) (PMMA) cements [9] or polyurethane scaffolds [10]. However, there are some drawbacks connected with this approach as well, because of the invasive surgical implantation procedure and poorly defined period of Vanc delivery [11].

The system we suggest is based on a hydrogel injectable matrix—gellan gum. Gellan gum (a polysaccharide, produced by an aerobic fermentation of carbohydrates by *Shingomonas elodea*) forms a threefold double-helical association. A ‘true gel structure’ of gellan gum is formed by the aggregation of these associations and is mediated by monovalent and/or divalent cations (e.g. Ca^2+^) [12]. Gellan gum has already been processed into mucoadhesive beads with ketoprofen [13], particles with ciprofloxacin for dermal applications [14], or as a composite with bioglass for bone tissue regeneration [15]. In this study we postulate that thanks to the hydrogel-based structure, the system will remain precisely at the site of infection, without spreading to adjacent areas.

To provide the hydrogel with a therapeutic function, the matrix was enriched with Vanc in two forms: dissolved and encapsultaed in poly(lactide-*co*-glycolide) (PLGA) nanoparticles. We postulated that such a combination would ensure the proper drug release profile, i.e. a burst release necessary to eliminate bacterial biofilm followed by a prolonged sustained release, to prevent its reconstitution. PLGA was selected as a material to fabricate Vanc-loaded nanoparticles, because it is bioresorbable, approved by Food and Drug Administration as well as European Medicines Agency and its degradation rate can be finely adjusted (i.e. by changing lactide to glycolide ratio, molecular weight and chain structure) [16]. PLGA’s utility for biomedical purposes has already been confirmed by its broad medical application in the form of sutures, screws or plates for osteosynthesis.

In general, our study aimed to fabricate an alternative solution for osteomyelitis treatment using a low-invasive procedure. We hypothesized that the material thanks to the hydrogel structure would be easy to administer (injectable) and thanks to addition of Vanc in two forms (dissolved and encapsulated) would ensure site-specific and well tailored drug delivery. The system composed was cross-linked to provide the injection with proper compactness and elasticity. We thoroughly assessed its handiness, accuracy of dosing, drug release kinetics and mechanical properties. Lastly we evaluated antimicrobial response towards the bacterial strains gathered form osteomyelitic joints as well as cytocompatibility in contact with osteoblast-like cells.

## Materials and methods

### Materials

Vancomycin hydrochloride, gellan gum (GelzanTM, low-acyl form, MW 200-300 kDa) and polyvinyl alcohol (PVA, Moviol 40-88), all of analytical grade, were obtained from Sigma-Aldrich, Poland. Mercaptoethanol, methanol, ethanol, isopropanol, orthophthaldialdehyde (OPA), phosphate-buffered saline concentrate (PBS buffer) and CaCl_2_ were all of analytical grade and were purchased from POCh, Poland. All the experiments were conducted in ultra high quality water (UHQ-water, produced on UHQ-PS, Elga, UK). Poly(lactide-*co*-glycolide) (PLGA, 85:15, Mn = 100 kDa, d = 1.9) was synthesized by a ring-opening polymerization in bulk at 100 °C using a biocompatible zirconium(IV) acetylacetonate initiator [17] in the Centre of Polymer and Carbon Materials of the Polish Academy of Sciences in Zabrze, Poland.

### Vancomycin-loaded nanoparticles fabrication

PLGA nanoparticles with vancomycin (NPs) were fabricated using a double emulsification/solvent evaporation technique. 10 mg of antibiotic (solid—S) was ultrasonicated (3 min, 40 % of the cycle, Sonics VibraCellTM, USA) in a 3.34 % w/v solution of PLGA in dichloromethane (3 ml, oil—O), producing the primary dispersion (S/O) on ice. The primary dispersion was then added drop by drop into 15 ml of 4 % w/v PVA solution on ice during ultrasonication and formed a secondary phase (S/O/water). Subsequently, the emulsion was stirred overnight (500 rpm) to remove dichloromethane. The dispersion of nanoparticles was centrifuged (14,000 rpm, 4 °C, 20 min) and flushed three times with UHQ-water. Obtained NPs were freeze-dried for 48 h and stored at 4 °C. Before further analyses, the particles were redispersed in UHQ-water using ultrasonication.

### Size and zeta potential of nanoparticles

The size, the polydispersity and zeta potential of NPs were determined by dynamic light scattering (DLS) and capillary electrophoresis measurements using a Zetasizer Nano ZS (Malvern Instruments) at 25 °C.

### Drug encapsulation and loading efficiencies

To determine the amount of encapsulated Vanc, the supernatant obtained after NPs centrifugation was gathered and examined fluorescently (λ_ex._—340 nm, λ_em._—455 nm; FLUOstar Omega, BMG Labtech) using the OPA assay [18]. Encapsulation (*%EE*) and loading efficiency (*%LE*) were determined according to the following equations:

1 $$\% EE = mass \, of \, Vanc \, in \, nanoparticles/initial \, mass \, of \, Vanc \, in \, the \, system \times 100\%$$

2 $$\% LE = mass \, of \, Vanc \, in \, nanoparticles/mass \, of \, nanoparticles \times 100\%$$

### Morphology of nanoparticles

Morphology of NPs was investigated using atomic force microscopy (AFM). A microscopic glass slide (Thermoscientific, Menzel-Glaser, Germany) was hydrophilized by 10 min sonication in ethanol followed by 12 h drying at room temperature and a drop of 0.1 % w/v suspension of NPs in UHQ-water was placed on it. Topographic images were recorded in contact mode on an Explorer AFM (Thermomicroscopes, spring constant *k* = 0.02 N/m, image size 3 μm × 3 μm). All the images were processed using SPMLab6.02 provided by the AFM manufacturer.

### Preparation of hydrogel-based injectable system

A 1.4 % (w/v) solution of gellan gum was prepared in UHQ-water at 90 °C. After the temperature was decreased to 50 °C, a 1.0 % w/v suspension of NPs (encapsulated antibiotic 0.09 % w/v) and dissolved antibiotic (free antibiotic 0.1 % w/v) were added. Final antibiotic content in the sample was 0.19 % w/v. To adjust the injectability, the samples were cross-linked by 0.3 % (w/v) CaCl_2_ and denoted as GG-Vanc-NPs. Reference samples were gellan gum with 0.3 % (w/v) CaCl_2_ (denoted as GG) and gellan gum with 0.3 % (w/v) CaCl_2_ and dissolved antibiotic (denoted as GG-Vanc). In all the samples the mass percentage of gellan gum was 0.7 % (w/v).

### Injectability

The injectability of GG and GG-Vanc-NPs samples was studied by the method described by Ghadhri et al. [19]. Force curves and the maximal force (F_Max_) needed to expel 1 ml of the samples from the 3 ml syringe with a standardized needle (18G, 0.85 mm inner diameter and 1.45 mm outer diameter, Becton Dickinson) were measured in a compression test (Zwick 1435, Germany). The crosshead speed of the testing machine was 50 mm/min.

### Rheological characteristics

Rheological tests were performed for GG and GG-Vanc-NPs (Physica MCR 501 Rheometer, Anton Paar, Graz, Austria; Couette CC10/T200 coaxial geometry with a bob diameter 10.002 mm, cup diameter 10.845 mm). To avoid evaporation, a layer of paraffin oil was applied. Gelation was analyzed by measuring storage and loss moduli (*G’* and *G”*) as a function of time during application of oscillatory sinusoidal deformation (0–30 min, frequency *f* = 1 Hz, strain *c* = 0.01 %). Then (30–60 min) the gels were broken by increasing strain (*c* = 0.01–100 %, *f* = 1 Hz). In the last time interval (60–90 min) behavior of gel after structures’ disruption was analyzed (*f* = 1 Hz, *c* = 0.01 %). All measurements were performed at 37 °C.

### Drug release experiment

The release profiles of drug from NPs and GG-Vanc-NPs, were analyzed. Dialysis bags (ZelluTransRoth, MWCO 12 kDa) were filled with 1 ml of NPs suspension (10 mg NPs, containing in total 0.9 mg drug) or 1 ml of GG-Vanc-NPs (containing in total 1.9 mg drug) and immediately immersed in the vials with 20 ml of phosphate buffered saline (PBS) at pH 7.2 and stirred (50 rpm) at 37 °C. At predetermined time intervals, 0.5 ml of PBS were collected for up to 40 days of the experiment and the amount of drug was measured with an OPA reagent [18].

### Antimicrobial activity

In order to check antimicrobial activity of GG-Vanc-NPs, agar diffusion tests were performed according to the Kirby-Bauer method. Growth inhibition against Gram-positive bacteria *Staphylococcus aureus* (SA1-KCR) and *Staphylococcus epidermidis* (SE1-KCR) isolated from infected joints (Krakow Centre of Rehabilitation and Orthopedics, 2012) was observed. Reference strains were *S. aureus* DSM 24167 (Deutsche Sammlung von Mikroorganismen und Zellkulturen) and *S. epidermidis* ATCC 700296 (American Type Culture Collection). Tested strains were incubated in 5 ml of Bacto™ Tryptic Soy Broth (Becton Dickinson) for 16 h at 37 °C and prepared at a concentration of 0.5 on the McFarland scale (1.5 × 10^8^ CFU/ml) in 0.7 % w/v NaCl solution. Then, the bacteria were seeded on Mueller–Hinton agar plates (Difco). In the left part of each plate a 3 mm well was made with a hole punch into which a tube-shaped GG-Vanc-NPs (2 mm height, 3 mm diameter) was introduced. In the right part a similar GG-Vanc-NPs sample was placed. As positive control vancomycin discs (30 µg, Oxoid, UK) were applied, while as negative control GG samples were tested. The plates were then incubated at 37 °C for 18 h and the zone of microbes growth inhibition (mm) was measured using Calibrating Viewer.

### Biological tests with osteoblast-like MG-63 cells

Gellan gum solution was autoclaved, CaCl_2_ and Vanc solutions were sterile-filtered and NPs were treated with UV-light for 40 min. Cytocompatibility was analyzed by the metabolic activity of MG-63 cells (European Collection of Cell Cultures, Salisbury, UK) incubated in the extract from samples done in Eagle’s minimal essential medium (EMEM, PAN BIOTECH, Germany) supplemented with 10 % fetal bovine serum, 1 % penicillin–streptomycin, 0.1 % sodium pyruvate (PAA, Austria). Cells cultured in pure EMEM acted as a control. Extract was obtained by incubation tube-shaped samples in EMEM for 24 h at 37 °C (0.1 g of material per 1 ml of medium). The extract was diluted in EMEM by factors of 1/1 (undiluted), 1/2 and 1/4. MG-63 cells (1.5 × 10^4^ cells/cm^2^) were cultured in a 48-well plate (Nunclon) for 24 h. Afterwards the medium was changed for extracts (1 ml) at the aforementioned dilutions. After another 24 h and 6 days metabolic activity was measured via reduction of resazurin in Alamar Blue reagent (10 % (w/v) Resazurin solution in PBS, Sigma-Aldrich). 0.05 ml of reagent was added, cells were incubated for 4 h at 37 °C and then the reduction was measured via fluorescence (λ_ex._—530 nm, λ_em._—590 nm; FLUOstar Omega, BMG Labtech) and calculated as follows:

3 $$\% \, Reduction \, of \, Alamar \, Blue \, = \left( {S^{x} - S^{control} } \right)/\left( {S^{100\% reduced} - S^{control} } \right) \times 100\%$$

where S^x^ is fluorescence of samples, S^control^ is fluorescence of EMEM without cells, S^100 %reduced^ is fluorescence of reagent reduced in 100 % (reagent with EMEM was placed in autoclave for 15 min at 121 °C).

Cell attachment, distribution and viability were evaluated on day 1 and 6 after live/dead staining (calcein AM/propidium iodide, Sigma-Aldrich according to the manufacturer’s protocol) using a fluorescence microscope (Axiovert 40, Carl Zeiss, Germany).

All results were expressed as a mean ± standard error of the mean (S.E.M.) from 3 individual measurements performed in triplicate (n = 9). Statistical analysis was performed using the unpaired *t-*test. Significant differences were assumed at *p < 0.05; **p < 0.01; ***p < 0.001.

## Results

### Properties of vancomycin-loaded nanoparticles

**Fig. 1:**
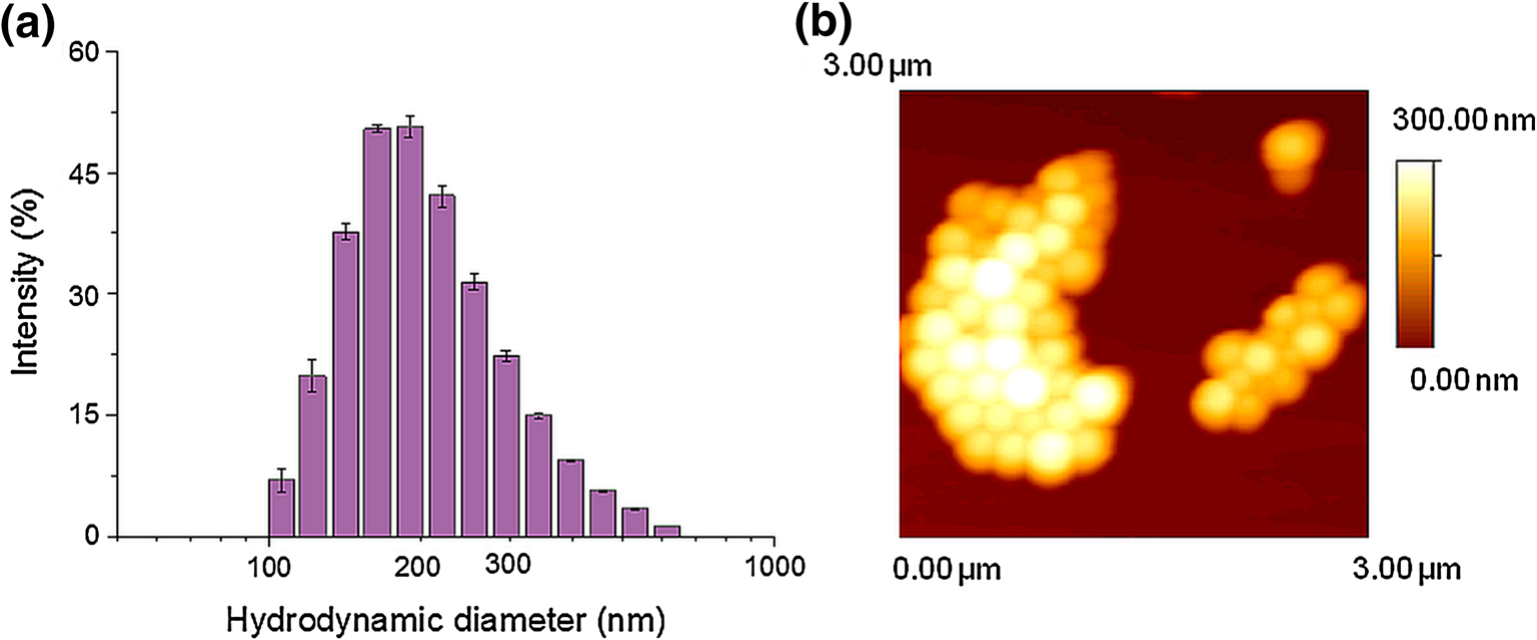
Histogram presenting hydrodynamic diameter of NPs obtained with Malvern Zetasizer NanoZS (**a**), AFM topographical image of NPs (**b**)

NPs produced with a double emulsification (S/O/W) were formed with average diameter 258 ± 11 nm and polydispersity index 0.240 ± 0.003. The diameter and polydispersity of empty nanoparticles were 234 ± 4 nm and 0.290 ± 0.029, respectively. A histogram presenting the hydrodynamic diameter of NPs is shown in Fig. 1a. Electrophoretic measurements showed that NPs had a negative surface charge with a zeta potential of −25.9 ± 0.2 mV; for comparison empty nanoparticles had zeta potential of −30.5 ± 0.6 mV. AFM analysis confirmed that NPs were smooth and spherical (Fig. 1b). The encapsulation and loading efficiencies of Vanc in the NPs were 55.2 ± 0.5 and 8.8 ± 0.1 %, respectively.

### Injectablity

**Fig. 2:**
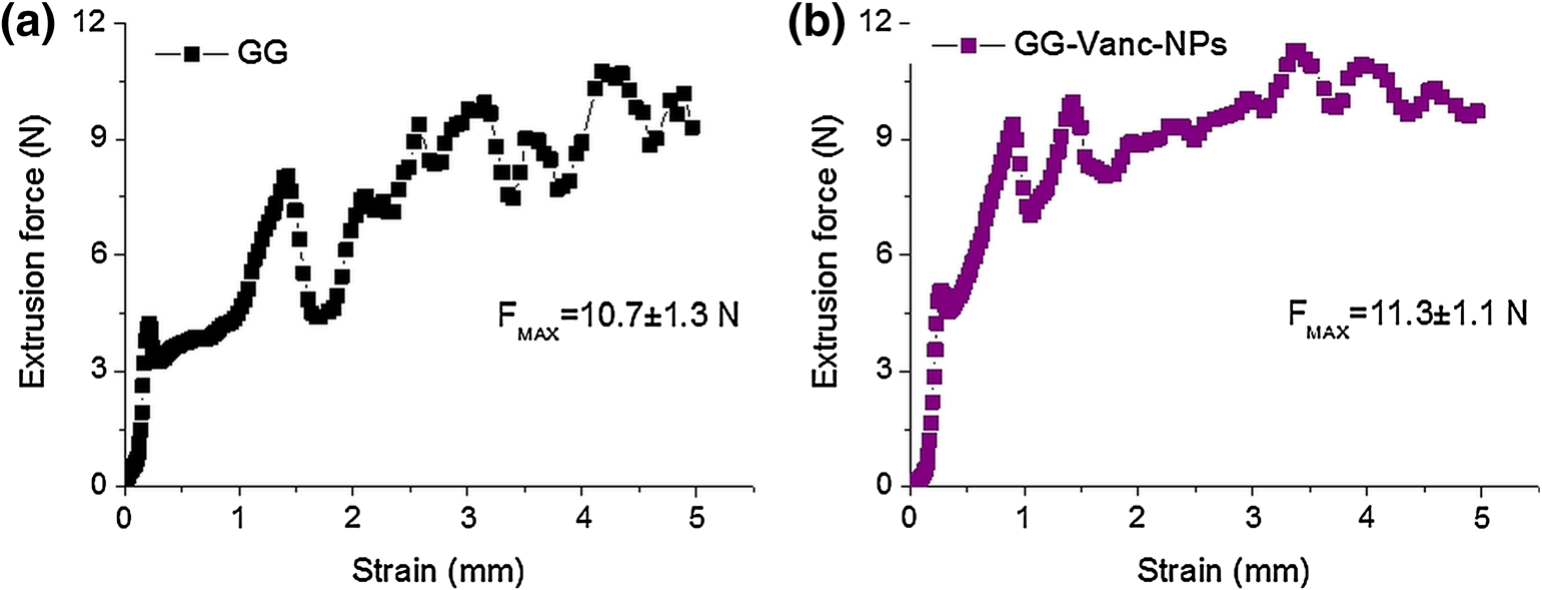
Force changes as a function of piston distance during injection of GG (**a**) and GG-Vanc-NPs (**b**); the values of maximal extrusion force (F_Max_, mean ± SEM) are also shown below the curves

To start extrusion of GG a force of at least 4.5 N was needed and during expulsion the force changed from 4 to 11 N (Fig. 2a). Regarding extrusion of GG-Vanc-NPs, the force needed to initiate the movement of the piston was ca. 5 N, whereas the force needed to continue the extrusion was slightly higher and less variable (7–12 N), and no blocking of the needle was observed (Fig. 2b). Maximal extrusion force was 10.7 ± 1.3 and 11.3 ± 1.1 N for GG and GG-Vanc-NPs, respectively.

### Rheology assay

Gelation took 7 and 22 min for reference and loaded system, respectively, and afterwards plateau phase was reached. Data regarding rheological properties in plateau (after leveling off) for GG and GG-Vanc-NPs showed significantly higher storage modulus (*G’*) than loss modulus (*G”*) value during and after gelation (Table 1, Assembling). The structure was much more elastic than viscous and exhibited the ability to self-organize after disruption by strain sweep deformation. After breaking, the moduli returned to initial relation (*G’* > *G”*) and values were similar to those in the gelation stage (Table 1, Reassembling).

**Table 1:** Rheological characteristics of GG and GG-Vanc-NPs. Storage modulus *G’* and loss modulus *G”* values obtained during gelation (assembling) of the matrix (*f* = 1 Hz, *c* = 0.01 %) and reassembling of the matrix after breaking by strain deformation (*f* = 1 Hz, *c* = 0.01 % – 100 %); n = 3

Sample	Assembling		Reassembling after breaking	
	*G’* (kPa)	*G”* (kPa)	*G’* (kPa)	*G”* (kPa)
GG	8.5 ± 0.9	0.5 ± 0.1	7.1 ± 0.7	0.5 ± 0.1
GG-Vanc-NPs	7.4 ± 1.5	0.4 ± 0.2	6.6 ± 0.9	0.4 ± 0.1

### Vancomycin release kinetics

**Fig. 3:**
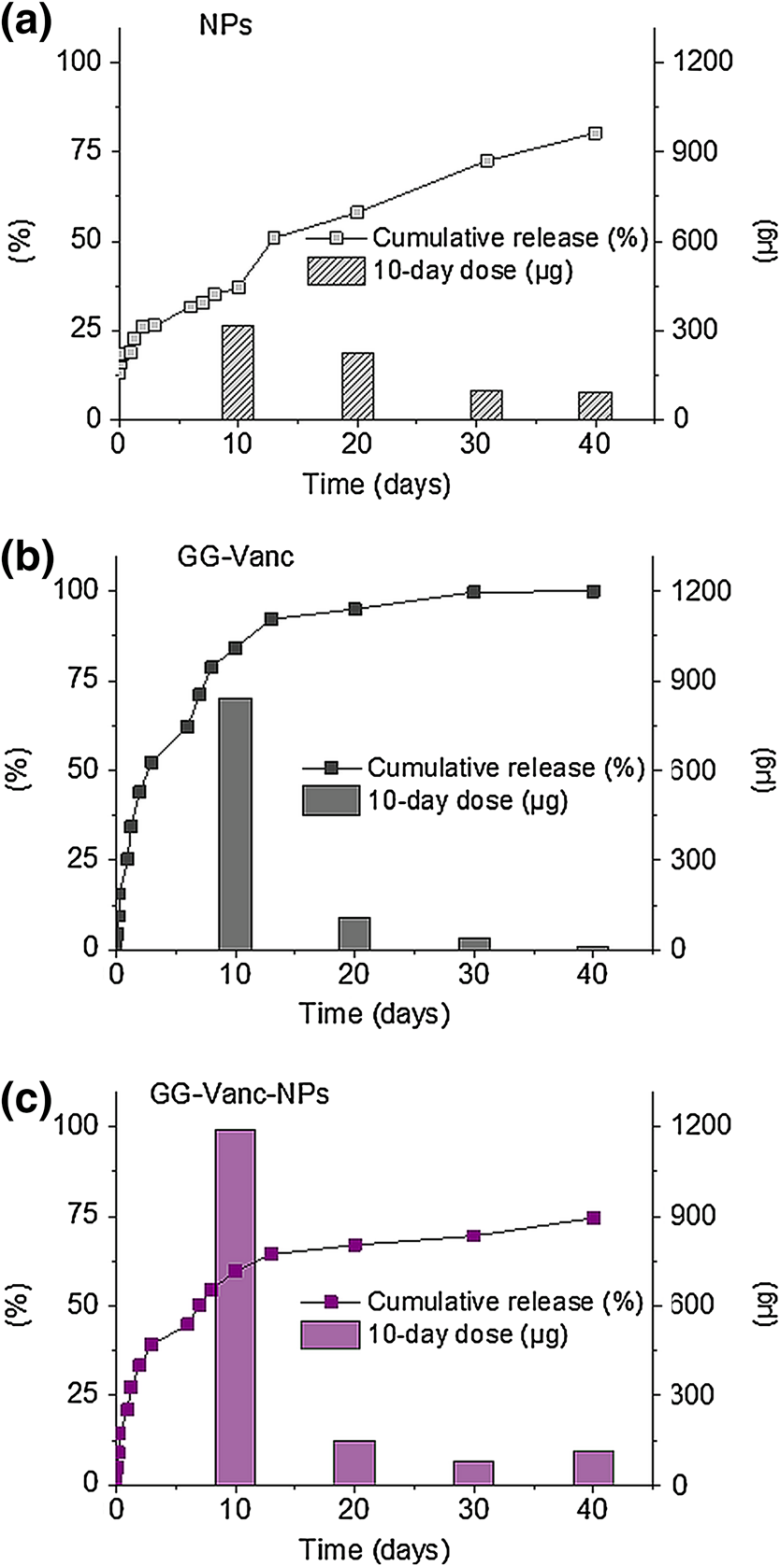
Vanc release profiles from **a** Vanc-loaded nanoparticles (1 ml of 0.1 % w/v NPs suspension, antibiotic content 0.9 mg) **b** gellan gum with antibiotic (1 ml of GG-Vanc, antibiotic content 1.0 mg) and **c** injectable system (1 ml of GG-Vanc-NPs, antibiotic content 1.9 mg). Merged pictures presenting cumulative release (*left axes, squares*) and 10-day doses (*right axes, bars*)

For NPs (Fig. 3a) an initial burst release on day 1 amounting to ~260 μg was measured, which was ~26 % of the total encapsulated antibiotic. Thereafter, the delivery was more sustained: 220–100 μg/10-day interval (in total ~83 % of the whole Vanc dose was liberated within 40 days). For GG-Vanc (Fig. 3b), a burst release after day 1 was also observed, which amounted to ~340 μg (~34 % of total encapsulated antibiotic). Thereafter, the release kinetics were less sustained than for NPs: the delivered amount varied between 110 and 10 μg/10-day interval and the whole Vanc dose almost exhausted up to day 20. For GG-Vanc-NPs (Fig. 3c) a burst release of ~900 μg (~47 % of Vanc content) was obtained that was followed by balanced delivery of ~120 μg/10-day interval. Up to day 40 a sustained delivery phase was observed. In total, the system delivered ~1.5 mg, i.e. ~80 % of Vanc.

### Activity against *Staphylococcus* spp.

**Fig. 4:**
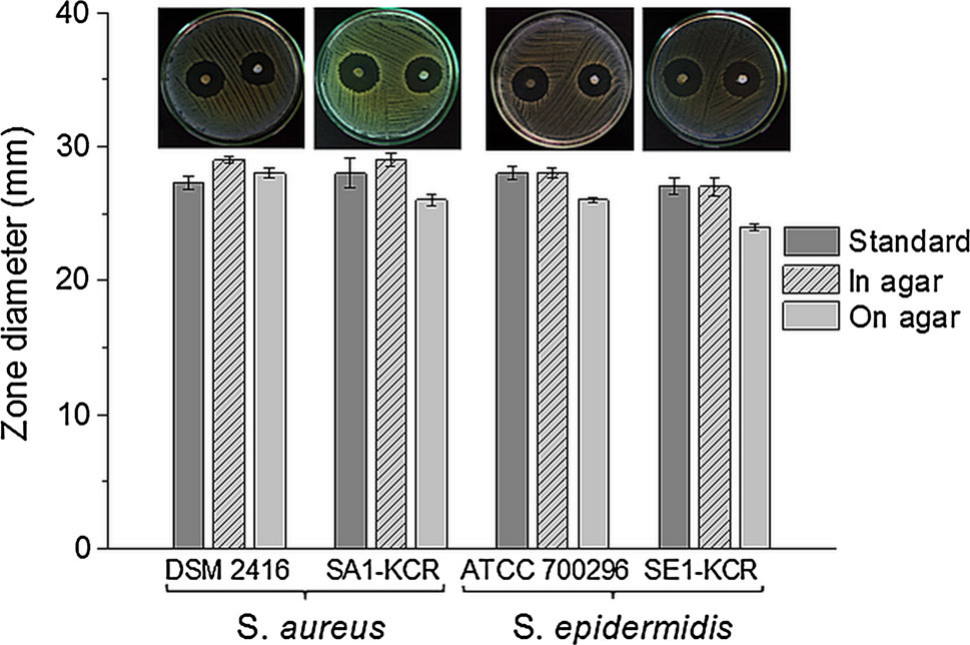
Inhibition of *Staphylococcus* spp. (DSM 24167—reference strain of *S.* *aureus*, SA1-KCR—isolate of *S.* *aureus* from infected joint, ATCC 700296—reference strain of *S. epidermidis*, SE1-KCR—isolate of *S. epidermidis* from infected joint) grown with GG-Vanc-NPs. Microphotographs were taken on Petri dish (diameter 9 cm), with a digital camera Olympus; the samples were introduced inside the 3 mm agar well or placed directly on the agar, for the *left* and *right* area of the Petri dish, respectively.  Mean ± SEM

In Fig. 4 inhibition zones and microphotographs of agar plates with *Staphylococcus* spp. cultured with GG-Vanc-NPs are presented. The diameters obtained were in the range 24.1–29 mm (depending on the way the samples were placed and type of the strain). For the vancomycin standards (30 µg) the zone diameter was 27–28 mm. For GG sample growth of *Staphylococcus* spp. was not inhibited (data not shown).

### Cytotoxicity with MG-63 cells

**Fig. 5:**
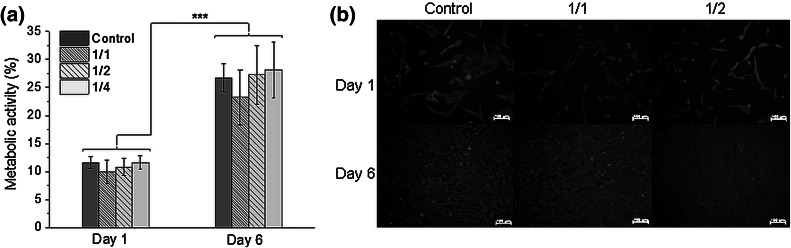
Metabolic activity of MG-63 cells evaluated by reduction of Alamar Blue (**a**) and live/dead staining (**b**) on 1 day or day 6 in contact with GG-Vanc-NPs extracts diluted in EMEM by the factors of 1/1, 1/2 or 1/4 and pure EMEM—Control; *green* are viable cells, *red* are dead cells (Olympus Carl Zeiss Axiovert, Germany). *Scale bar* 100 μm. Mean ± SEM; ***p < 0.001 (Color figure online)

Metabolic activity studies performed on MG-63 osteoblast-like cells on day 1 and 6 showed that cell function was not significantly altered by incubation in GG-Vanc-NPs extracts (Fig. 5a). Regarding more concentrated extracts, a slight tendency to down regulate cells’ activity was seen, but the differences were not significant. On day 6 the cells increased their activity significantly as compared to day 1.

For all the samples at day 1 (Fig. 5b, upper row) the cells showed spindle-shape or spread morphology resembling that under pure cell culture media conditions (Control); most of the cells were green and viable and only some dead cells were observed. At day 6 the cells were viable, formed a monolayer and exhibited a high level of metabolic activity.

## Discussion

This study aimed to develop a new injectable system with Vanc for osteomyelitis treatment. The hydrogel-based structure of the injection contained a dissolved portion of antibiotic, antibiotic-loaded nanoparticles and a cross-linker (Ca^2+^) that by ionotropic gelation ensured elasticity, compactness and stability of the whole structure. The system was characterized from the point of view of surgical handling, precision of dosage, mechanical stability, rheology and drug release kinetics. For in vitro evaluation, the biological response of pathogens causing osteomyelitis (*Staphylococcus* spp.) and osteoblast-like MG-63 cells were investigated.

### Vanc-loaded nanoparticles

Vancomicin is a model water-soluble antibiotic, widely used in osteomyelitis treatment and prevention of osseous staphylococci-derived infections [20]. However in order to effectively encapsulate Vanc in a hydrophobic matrix (e.g. PLGA) its hydrophilic character is an obstacle to achieve high solubilization efficiencies. A rapid partitioning into the external water phase occurred and finally 55.2 ± 0.5 and 8.4 ± 0.1 % of encapsulation and loading efficiencies, respectively, were obtained. Our results are similar to those obtained in other studies on vancomycin-loaded PLGA particles of submicron size produced via double emulsification [21]. The negative charge of PLGA nanoparticles (−25.9 ± 0.2 mV) was due to ionization of carboxylic end groups of the polymer and may be interpreted as an indicator of suspension stability: the repulsion occurring prevented phase separation due to aggregation [22]. The batch of NPs also displayed low polydispersity (see Fig. 1a, b).

### Handling of injectable system

Currently the low-traumatic therapies are strongly recommended [23]. Here, great attention is paid to injections that are site specific, easy to use and precisely fill the bony defect [24]. In this study we showed that to sustain the GG extrusion a variable pressure (reflected as force changes) was needed (see Fig. 2a). This observation can be connected with spatial inhomogeneities that result from non-uniform distribution of cross-links arising during ionotropic gelation (the higher the number of cross-links, the more diversified the structure) [25]. Presumably, such differences in forces applied on the piston resulted from such structure disorders.

On the other hand, GG loaded with nanoparticles and dissolved drug was more compact, bulky and exhibited a paste-like character. The pressure necessary to initiate and continue extrusion was slightly higher, but less variable, enabling much more precise dosing.

According to recommendations the maximal extrusion force (a measure of surgical handling) should be lower than 20 N [26]. For GG-Vanc-NPs, an extrusion force of 11.3 ± 1.1 N was measured which confirmed its easy handling during application. The values were comparable to previously used medical materials such as fillers for craniofacial surgery (Restylene, Therafill, *F*_*MAX*_ = 10–12 N) [27], tricalcium phosphate pastes intended for bone filling (5.4 ± 1.1 to 18.5 ± 1.7 N) [28] or injectable hydrogel-based systems for local delivery of bisphosphonates (9.5 ± 1.0 N) [29].

### Rheological properties

Performing rheology studies enables understanding of the development of structural alterations of parenteral formulations during handling or administration, i.e. syringe filling or injection [30]. Gellan gum natively follows cation-mediated aggregation of the triplets of double helices into brittle gel [12]. The character and mechanical properties of the system were analyzed by a strain sweep deformation test. It was observed that both samples were much more elastic than viscous (more rubbery-like than fluid-like). NPs’ addition neither significantly decreased mechanical properties nor the ability to self-assemble after disruption (transformation of gel to sol was not obtained). The moduli values immediately restored the initial state, i.e. significant domination of *G’* over *G”*. Such behavior, i.e. electrostatically-driven immediate structure arrangement after destruction, was previously described by Wang et al. for colloidal gels obtained from gelatin nanospheres bearing opposite charges [31].

### Drug release profile

The antibiotic release kinetics from typical materials of biomedical use (e.g. PMMA cements) are poorly characterized. Cumulatively only 25 % of the drug is released, whereas 60 % of the liberated dose is due to burst release. As a result, very quickly (within 1**–**2 weeks) the drug concentration falls well below therapeutic level [10, 32]. In general there is a recommendation to deliver high doses initially to clear tissue from bacterial biofilm, that should be followed by sustained and prolonged delivery in order to prevent microbial repopulation [32, 33].

For NPs (see Fig. 3a) we observed: (i) a burst phase, (ii) a slowdown around day 10 and (iii) a fine, sustained delivery of medium doses in the following 10-day intervals. The first phase can be connected with diffusion of the drug located in the subsurface region of NPs [34]. The slowdown in drug release on day 10 possibly was related to depletion of surface-associated drug [34]. In the last phase the release dose was higher but constant. Possibly along with degradation, porosities and channels accessible for water were created (the morphology studies for NPs showed an initially smooth surface without cavities) that facilitated elution of the hydrophilic drug out of NPs, indicating an erosion-diffusion drug release mechanism, as shown in other studies [34]. Although the drug release profile from NPs seems to comply with the demands of the antimicrobial therapies, the use of unprocessed NPs is rather questionable due to possible phagocytic uptake [35].

For GG-Vanc the cumulative release curve (see Fig. 3b) showed rapid release, slowing down with time, and soon depletion of Vanc 10-day doses. Possibly it was caused by fast leaching out of Vanc from GG structure, which shows a mainly diffusion-controlled manner of release enhanced by swelling [36]. Although the gel structure ensured deformability and injectable properties, due to lack of a sustained release phase its applicability in osteomyelitis treatment is less advantageous.

The best results were observed for GG-Vanc-NPs (see Fig. 3c)—the system exhibited both a very prominent burst phase as well as a fine prolonged phase of sustain delivery. Possibly, the mechanism of drug release in this case is a combination of erosion-diffusion release enhanced by hydrogel swelling, which are both of key importance from the point of view of therapeutical success.

### Antimicrobial potential and cytocompatibility

To monitor antimicrobial activity both clinical isolates taken from osteomyelitic joints and reference strains were used. The latter are equally important to study selection evasion of prophylaxis by tolerant strains or interspecies transfer of antimicrobial resistance genes in the microenvironment of a biofilm [37]. Zones of inhibited growth 24–29 mm in diameter were obtained (see Fig. 4). Larger zones occurred for GG-Vanc-NPs placed inside the agar well than directly on the agar surface; after placement in the well the higher contact area of the sample seemed to facilitate drug transport. In other studies on polyurethane scaffolds for bone wounds antimicrobial protection inhibition zones of ca. 20 mm were obtained [10]. It can be concluded that antibiotic released from GG-Vanc-NPs preserved its antimicrobial activity, so processing did not alter the pharmacological efficiency of Vanc. In vitro tests performed in contact with MG-63 cells showed that the system was cytocompatible with bone-forming cells (see Fig. 5a, b).

## Conclusion

The system based on vancomycin loaded nanoparticles and vancomycin embedded in gellan gum matrix: (i) was easy to apply (injectable), (ii) showed good mechanical properties (self-assembled after disruption by strain deformation), (iii) ensured local antibiotic delivery with an advantageously high burst phase and a long sustained phase, (iv) demonstrated antimicrobial activity against *Staphylococcus* spp. and (v) was cytocompatible with osteoblast-like cells. The system demonstrated potential for the intrabone antibiotherapy of osteomyelitis.
